# Impact of Educational Interventions on Knowledge About Hypertensive Disorders of Pregnancy Among Pregnant Women: A Systematic Review

**DOI:** 10.3389/fcvm.2022.886679

**Published:** 2022-06-20

**Authors:** Kosar Gholami, Narges Norouzkhani, Meraj Kargar, Hamidreza Ghasemirad, Atieh Jafarabadi Ashtiani, Shamim Kiani, Mahdi Sajedi Far, Maryam Dianati, Yasaman Salimi, Amirmohammad Khalaji, Sara Honari, Niloofar Deravi

**Affiliations:** ^1^Student Research Committee, Semnan University of Medical Sciences, Semnan, Iran; ^2^Department of Medical Informatics, Faculty of Medicine, Mashhad University of Medical Sciences, Mashhad, Iran; ^3^Student Research Committee, Afzalipour Faculty of Medicine Kerman University of Medical Sciences, Kerman, Iran; ^4^Student Research Committee, Shahid Sadoughi University of Medical Sciences, Yazd, Iran; ^5^Faculty of Medicine, Shahed University, Tehran, Iran; ^6^Student Research Committee, Department of Midwifery, Faculty of Nursing and Midwifery, Ahvaz Jundishapur University of Medical Sciences, Ahvaz, Iran; ^7^Student Research Committee, Rafsanjan University of Medical Sciences, Rafsanjan, Iran; ^8^Student Research Committee, School of Dentistry, Kermanshah University of Medical Sciences, Kermanshah, Iran; ^9^School of Medicine, Tehran University of Medical Sciences, Tehran, Iran; ^10^Faculty of Medicine, Mashhad University of Medical Sciences, Mashhad, Iran; ^11^Students Research Committee, School of Medicine, Shahid Beheshti University of Medical Sciences, Tehran, Iran

**Keywords:** hypertensive disorders of pregnancy, preeclampsia, education, hypertension, pregnancy

## Abstract

**Background:**

Hypertensive disorders of pregnancy (HDP), including chronic hypertension, preeclampsia and gestational hypertension, is the cause of about 50,000 deaths out of 400,000 perinatal deaths. HDP is an effective risk factor in stroke, type 2 diabetes, and cardiovascular diseases like ischemic heart disease. There is a significant relation between HDP, lifestyle, and knowledge. Unfortunately, many studies showed that pregnant women have lack of knowledge about HDP. Therefore, the importance of educational interventions is, today, more acknowledged than before.

**Aim:**

The goal of this systematic review was to investigate the effect of interventional educations on the knowledge of pregnant women about HDP.

**Methods:**

A systematic review of the related articles was conducted. We included English randomized controlled trials published up to December 2021, including pregnant women as population, HDP as the outcome, and educational interventions as the intervention.

**Results:**

After the process of study selection, six articles containing 819 pregnant women were included in this study. Educational pamphlets, mobile-based application, a mixture of pamphlets, pictographic magnet and videos, and a combination of PowerPoint and data show projectors and conversation were the educational interventions in these studies.

**Conclusions:**

The positive effects of educational interventions on the knowledge of women with HTP were observed in all studies. The higher knowledge leads to HDP-related complications.

**Systematic Review Registration:**

https://archive.org/details/osf-registrations-gcs5r-v1, identifier: doi: 10.17605/OSF.IO/GCS5R.

## Introduction

Hypertensive disorders of pregnancy (HDP) is observed in 5–10% of pregnant women worldwide (1). Chronic hypertension, preeclampsia, and gestational hypertension are the three types of HDP. Chronic hypertension, as a hypertension diagnosed before pregnancy or before the 20th week of pregnancy, may convert to preeclampsia. Gestational hypertension, occurred after 20 weeks of pregnancy, can also lead to preeclampsia (2). Preeclampsia is a hypertensive disorder characterized by proteinuria and the onset of hypertension beginning after 20 weeks of pregnancy (3). More than 50,000 deaths of mothers and 400,000 of perinatal deaths happen because of HDP, specially preeclampsia, each year (4, 5). The incidence of stroke and ischemic heart disease in women with preeclampsia is about 2.5 times higher than normal pregnancies (6, 7). The risks of renal disease and type 2 diabetes are also elevated by preeclampsia (8). In addition to preeclampsia, gestational hypertension and chronic hypertension are also long-term risk factors in cardiovascular disease (9). Nowadays, some screening programs are performed all over the world for the identification of women with the signs of preeclampsia. Cooperation of the women is one of the most important factors in the success of these programs. The cooperation is associated with the knowledge and the level of education of pregnant women (10, 11). Many studies found out that pregnant women had poor knowledge about increased cardiovascular risks after HDP (12). Pregnant women often do not participate in the programs for life-style changing because of low amount of knowledge, lack of suitable follow-up, and, also, the higher price of healthier food (13–15). Studies indicated that poor education levels of pregnant women led to dangerous conditions like pre-mature delivery or death of neonates (16). Researchers indicated that women were assessed for their cardiovascular disease less than men or the assessment was generally performed after the diagnosis among them (17). This makes the importance of knowledge of pregnant women about HDP more than before. The patient's knowledge plays an important role in preventing risk factors like cardiovascular disease, monitoring blood pressure, and helping the patient to know about the condition of severity of the disease, symptoms, and the management of them by a good lifestyle, including appropriate diet and lifestyle modifications (18). As noted, due to the importance of increasing the levels of education among pregnant women about HDP, different educational interventions were used in studies. Mobile-based applications, graphics-based educational tools, and pictorial cards are some of the examples (19–21). As far as we know, there are no presently available systematic reviews on the impact of educational interventions on knowledge of pregnant women about HDP. This review can help the medical staff and pregnant women for better choice of educational interventions to better manage HDP.

## Methods

This systematic review study was conducted in accordance with the Preferred Reporting Items for Systematic Reviews and Meta Analyses (PRISMA2020) Statement (22). In accordance with the PICO criteria, the “participants” were exclusive to pregnant women; the types of “intervention” covered were educational interventions; the “comparator” was not determined; and the “outcome” was knowledge about HDP. This review has been registered on The Open Science Framework (OSF) (Registration doi: 10.17605/OSF.IO/GCS5R available at https://archive.org/details/osf-registrations-gcs5r-v1).

### Search Strategy

**Figure 1:**
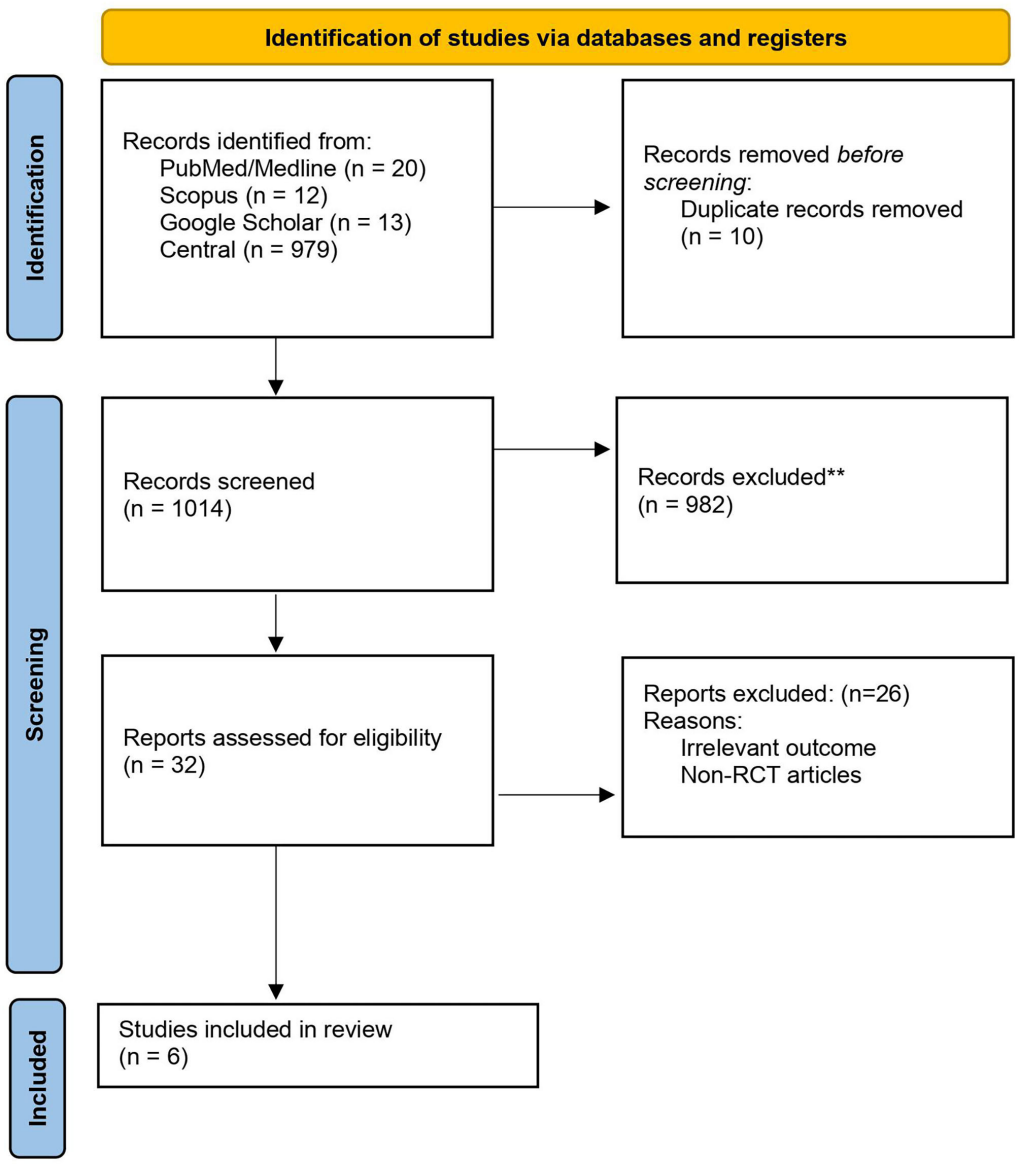
A PRISMA 2020 flow diagram.

We identified original RCTs through searching for English language articles published up to December 2021 in PubMed/MEDLINE, Scopus, Google Scholar, and Cochrane Central Register of Controlled Trials (CENTRAL) databases. Additionally, the duplicate records were removed using EndNote (v.7, Thomson Reuters, Toronto, Canada). Two reviewers (MD and KGh) developed the search strategy as followed: (“hypertension^*^” OR “hypertensive” OR “hypertensive disorders”) AND (“education^*^” OR “inform^*^” OR “knowledge”) AND (“pregnancy” OR “pregnant”). Table 1 shows the search strategies for PubMed/Medline, Scopus, and central databases. We also screened the references of relevant studies to identify eligible studies. The PRISMA flow diagram is available in Figure 1.

**Table 1:** Search strategies for PubMed, Scopus, and central databases.

**Search engine**	**Search strategy**	**Additional filters**
PubMed/MEDLINE	(education[tiab] OR educate[tiab] OR educational[tiab] OR inform [tiab] OR informative[tiab] OR “education” [Mesh] OR “educate”[Mesh] OR “educational” [Mesh] OR “inform” [Mesh] OR “informative”[Mesh]) AND (hypertension[tiab] OR hypertensive[tiab] OR “hypertension” [Mesh] OR “hypertensive”[Mesh]) AND (knowledge[tiab] OR “knowledge”[Mesh]) AND (pregnant [tiab] OR pregnancy[tiab] OR preeclampsia[tiab] OR Hypertension, Pregnancy-Induced[tiab] OR “pregnant”[Mesh] OR “pregnancy” [Mesh] OR “preeclampsia”[Mesh] OR “Hypertension, Pregnancy-Induced”[Mesh]) AND (intervention[tiab] OR “intervention” [Mesh])	**English, December 1** ^**st**^ **, 2021**
Scopus	(education* OR educate* OR educational* OR inform* OR informative*) AND (hypertension* OR hypertensive*) AND (knowledge*) AND (pregnant* OR pregnancy* OR preeclampsia* OR Hypertension, Pregnancy-Induced*) AND (Intervention*)	**English, December 2** ^**nd**^ **, 2021**
Central	#1: (Educate):ti,ab,kw OR (Educational):ti,ab,kw OR (Education):ti,ab,kw OR (inform*):ti,ab,kw OR (Knowledge):ti,ab,kw #2: MeSH descriptor: [Education] explode all trees #3: (Pregnant):ti,ab,kw OR (Pregnancy):ti,ab,kw OR (preeclampsia):ti,ab,kw #4: MeSH descriptor: [Pregnancy] this term only #5: (Hypertension):ti,ab,kw OR (Hypertensive):ti,ab,kw #6: MeSH descriptor: [Hypertension] explode all trees #7: #1 OR #2 #8: #3 OR #4 #9: #5 OR #6 #10: #7 AND #8 AND #9	**English, December 21** ^**st**^ **, 2021**

### Inclusion Criteria

All primary research studies that found the following PICOS criteria were included for review if:

(A) Population: pregnant women (P);
(B) Interventions: educational intervention (I);
(C) The control group: standard care or forfeiture of any intervention; if there was no control group (C);
(D) Type of the primary outcome: hypertensive disorders of pregnancy (O);
(E) Type of study design: English language RCTs (S).

### Data Screening and Extraction

Two reviewers (ShK and AJA) assessed and screened titles and abstracts to recognize related studies using a form developed by the research team. Full texts of studies were retrieved for “Yes” and “Maybe” assessment for eligibility study. We resolved discrepancies and disagreement by consensus. Data extraction was completed by two independent assessors (MS and HGh). Discrepancies were resolved by consensus and discussion between two reviewers.

### Quality Assessment of Included Studies

**Figure 2:**
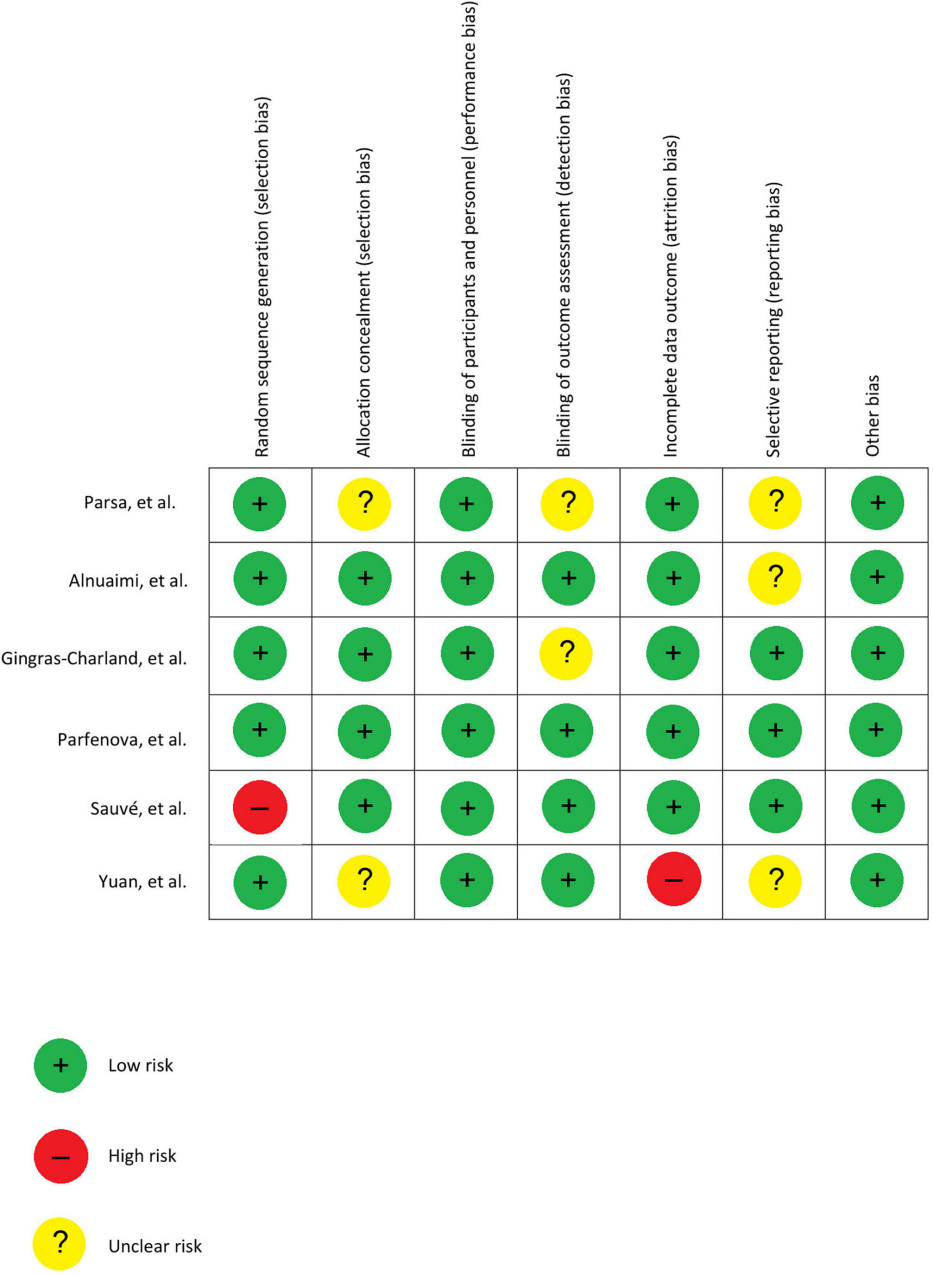
Risk of bias of the included RCTs.

For each study, two assessors (YS and KGh) independently assessed all included studies according to the Cochrane risk of bias tool (23). The Cochrane risk of bias tool is a standard and common tool that includes all the essential questions to determine and judge the methodological quality and the risk of bias focusing on 6 domains, including sequence generation, allocation concealment, blinding, in-complete data, and selective reporting, and other bias and disagreement were resolved by consensus and discussion *via* two assessors (Figure 2).

## Results

Through the process of selection, six articles were included in this study; the summary of the findings of included studies is summarized in Table 2. All of the included studies were randomized clinical trials. Three of the studies were performed in Canada. The other studies were conducted in Iran, Jordan, and China. These studies investigated the effect of educational interventions on some obstetrics and non-obstetrics outcomes, such as knowledge, Apgar score, systolic blood pressure (SBP), diastolic blood pressure (DBP), satisfaction, awareness, weight gain, and anxiety about hypertensive disorders of pregnancy (including pre-eclampsia and gestational hypertensive disease) among 910 pregnant women. The most frequent disorder was pre-eclampsia. Various educational intervention tools were used for assessment of efficacy of education in the included studies: educational pamphlets (27, 28), mobile-based educational application (11), a combination of pamphlets, pictographic magnet, and videos (24), prenatal health education and nutrition interventions (29), and a mixture of PowerPoint, as well as data, show projectors and conversation (26) were used for the purpose of education (Table 2). All studies reported a positive impact of educational interventions on the hypertensive disorders of pregnancy. Not only the educational interventions improved the obstetrics outcomes, but also the non-obstetric parameters were affected by them. Overall, the significant higher knowledge score (24, 26), greater levels of decreased DBP (26, 28), reduced SBP (28), the higher Apgar score (26) and satisfaction (26, 28), and more awareness about HDP complications (26) were observed in the included studies (The *p*-value of the mentioned outcomes was reported <0.05 in the articles).

**Table 2:** A summary of the findings of the included studies.

**First** **author**	**Year**	**Country**	**Type** **of study**	**Number of** **participants**	**Mean age** **intervention** **(±SD)**	**Hypertensive** **disorder of** **pregnancy**	**Educational** **intervention** **type**	**The time of** **intervention**	**Association of educational** **intervention on knowledge about hypertensive disorders** **of pregnancy**	**Obstetrics** **outcome** **overall**	**Obstetrics** **outcome** **(intervenrion)** **(±SD)**	**Obstetrics** **outcome** **(control)** **(±SD)**	***P*** **-value**	**Non** **obstetrics** **outcome**	**Obstetrics** **outcome** **(intervenrion)** **(±SD)**	**Obstetrics** **outcome** **(control)** **(±SD)**	***P*** **-value**	**Reference**
Parsa	2019	Iran	RCT	108	27.93±5.1	Pre- eclampsia	A mobile-based educational application	12 weeks	Yes	Knowledge about the symptoms, signs and complications of preeclampsia increased and adverse effects were reduced.	HDP Knowledge score: Before: 14.84±17.55 After: 78.08 ±14.19	HDPKnowledge score: Before: 14.56±17.55 After: 15.75±19.49	0.94	A mobile-based educational application improved health recovery and managing the condition, enhanced participation in preventive behaviors.	No statistics reported	No statistics reported	No statistics reported	(11)
Gingras-Charland	2018	Canada	RCT	362	28.9±.6	Pre- eclampsia	An informative pamphlet, a video, and a pictographic magnet	4 weeks	Yes	Significant increase in knowledge about risk factors, symptoms, complications for the fetus or newborn and complications for mother and treatments.	HDPKnowledge scores: 70.1±19.2	HDPKnowledge scores: 51.1±23.4	<0.001	Educational intervention raised satisfaction and had no significant effects on the level of anxiety.	Anxiety: 2.40 ± 1.07 Satisfaction : 5.1±0.7	Anxiety: 2.53 ± 0.95 Satisfaction: between 1.7 and 3.8	*P*-values Anxiety: <0.001 satisfaction: 0.007	(24)
Yuan P	2018	China	RCT	90	34.4±4.3	Gestational hypertensive disease	Prenatal health education and nutrition interventions (More emphasis is placed on using interpretative, encouraging, persuasive language to explain relevant knowledge to patients and clarify their necessity.)	Not reported	Yes	Systolic and diastolic blood pressure decreased significantly and adverse consequences were reduced.	Systolic blood pressure: 134±3 Diastolic blood pressure: 83±2	Systolic blood pressure: 148±5 Diastolic blood pressure: 95±1	*P* < 0.05	Prenatal health education and nutrition interventions caused weight gain of pregnant women, raised satisfaction, maintained personal health, improved comfort and prognosis and quality of life.	weight gain: Low level: 3(6.7%) Normal level: 40(88.9%) High level: 2(4.4%) Satisfaction: 93.3%	weight gain: Low level: 10(22.2%) Normal level: 24(53.3%) High level: 11(24.4%) Satisfaction 71.1%	*P-*values (weight and satisfaction) <0.05	(25)
K. Alnuaim	2020	Jordan	RCT	113	29.87	Pre- eclampsia	An interventional programabout preeclampsia on high-risk preeclampsia Jordanian women's awareness and pregnancy outcomes (using PowerPoint and a data show projector using interactive images and conversation to educate participants in each group about the selected topics)	12 weeks	Yes	Promote Apgar score/ significant decrease in diastolic blood pressure/ no significant differences in terms of gestational age, newborn weight, systolic blood pressure, mode of birth, NICU admission, mother newborn status (dead or alive) and the final diagnosis/ improve awareness about preeclampsia complication, risk factors, signs and symptoms, prevention, management and preeclampsia definition/ better perfusion and performance of placentas.	Apgar score (1st min): 6.82± 1.42 Apgar score(5th min):8.32±0.95 systolic blood pressure: 116 ±18.8 diastolic blood pressure: 74 ±11.4	Apgar score(1st min): 6.05±1.63 Apgar score(5th min): 7.82 ±1.31 systolic blood pressure: 117 ±19.2 diastolic blood pressure: 79±13.1	*p*-value apgar score(1st min): 0.008 *p*-value apgar(5 st min): 0.024 *p*-value systolic blood pressure : 0.942 *p*-value diastolic blood pressure : 0.019	Total awareness increased significantly in interventional group	Total awareness: Before: 13.228±5.19 After: 27.122 ± 4.157	Total awareness: Before: 11.267 ±9.27 After: 11.5 ± 8.115	*P*-value (total awareness)= 0.00	(26)
Nadine Sauvé, MD	2008	Canada	RCT	100	29.71	Pre- eclampsia	Educational pamphlet	Not reported	Yes	Significant increase in knowledge about risk factors, symptoms, fetal complications, maternal complications and treatment.	Knowledge about fetal complications: 94% Knowledge about Maternal complications of death : 84% Knowledge about delivery treatment : 90% Knowledge about Antihypertensive: 84%	Knowledge about fetal complications: 86% Knowledge about Maternal complications of death 41% Knowledge about delivery treatment : 78% Knowledge about Antihypertensive: 78%	*P*-value fetal complications : 0.19 *p*-value Knowledge about Maternal complications of death : <0.01 *p*-value Knowledge about delivery treatment : 0.09 *p*-value Knowledge about Antihypertensive: 0.39	Educational pamphlet decreased level of anxiety about baby's health, increased level of anxiety about mother's health, raised satisfaction.	Anxiety field of mother's health: 3.90 Anxiety field of baby's physical health: 4.70	Anxiety field of mother's health: 3.73 Anxiety field of baby's physical health: 4.87	*p*-value Mother's health: 0.54 *p*-value Baby's physical health: 0.52	(27)
Maria Parfenova, MD	2020	Canada	RCT	137	30.9	preeclampsia, eclampsia, gestational hypertension, and pre-existing hypertension	educational pamphlet	68 weeks	Yes	Knowledge about risk for future pregnancy improved and global knowledge score raised.	Global knowledge score (before): 69.4% Global knowledge score (after 1 months) :88.2% Knowledge about future pregnancy (before): 64.6% Knowledge about future pregnancy (after 1 months): 87.6%	Global knowledge score (before): 71.4% Global knowledge score (after 1 months) : 71.3% Knowledge about future pregnancy (before): 65.9% Knowledge about future pregnancy (after 1 months): 67.9%	*P*-value Global knowledge <0.0001	Educational pamphlet improved knowledge about risk for future health, increased risk perception of heart disease, but no difference for hypertension or stroke, raised satisfaction and had no effect on the level of anxiety.	Knowledge about risk perception of heart disease (before) : 4.0 ± 0.78 Knowledge about risk perception of heart disease (after): 4.3 ± 0.94 satisfaction: 5.6 ± 0.66 Anxiety (before): 3.7 ± 1.0 Anxiety (after): 3.8 ± 1.0	Knowledge about risk perception of heart disease (before) 3.9 ±1.14 Knowledge about risk perception of heart disease (after): 4.0 ± 0.97 satisfaction: 3.6 ± 1.33 Anxiety (before): 3.9 ± 1.1 Anxiety (after): 4.0 ± 1.0	*p*-value increased risk perception of heart disease: 0.036 *p*-value satisfiction < 0.0001 *p*-value anxiety : 0.6746	(28)

## Discussion

The present study reviewed research on the effect of educational intervention on pregnant women's knowledge about HDP (including chronic hypertension, preeclampsia, and gestational hypertension). All included studies showed that providing training related to HDP is effective in increasing pregnant women's knowledge about the disease. Accordingly, providing educational interventions through various methods, including mobile applications (11), pamphlets (24, 27, 28), face-to-face training approaches (25, 26), and a combination of different training methods, increases the knowledge of pregnant women in this regard.

The low mean score of pre-eclampsia knowledge before the educational intervention indicates a very poor perception of possible risks of elevated hypertension by pregnant mothers (11). So, increasing relevant knowledge results in early awareness about the signs and symptoms leading to timely referral to physicians which, in turn, followed by suitable care and treatment, which totally provide healthier outcomes for both mothers and babies (30). Evenly, this kind of intervention was considered as the fundamental element for the recovery of maternal hypertension, which prepares the healthy conditions for pregnant women (25).

A graphitic educational tool increased the knowledge of pre-eclampsia 8 and 22% compared with standard pamphlets and peers with no education, respectively (21). However, the impact of a mobile-based educational application on the knowledge of the subjects was considerably higher (11). This increased growth in the level of knowledge that is related to the type of educational intervention. Widespread use of smartphones, along with the ease of access to a variety of information through different applications, improves the process of training. Also, this approach is effective in maintaining the individuals' health, controlling the related condition, and preventing risky behaviors (31).

An educational program on pre-eclampsia increased the awareness of high-risk women significantly, which is reflected in improving certain pregnancy outcomes like Apgar scores and mean diastolic blood pressure. Intriguingly, self-monitoring, which is represented in adherence to the provided information and recommendations, was also increased upon improving awareness on pre-eclampsia. For instance, informed mothers control their mean diastolic blood pressure with more caution compared with those without educational intervention (26). In order to guarantee adherence and commitment of patients to a self-monitoring process, an educational program preferably includes physicians, obstetricians, and other health care providers (26).

Studies have shown that raising awareness is strongly associated with early detection of pregnancy risks, which, in turn, can prevent dangerous complications (21, 30). In a study from Ethiopia, Wassihun et al. (31) found that mothers who participated in antenatal care were 1.26 times more likely to be aware of the symptoms of labor risk than those who were not. It was also found that the participants who gave birth in specialized health centers were 3.57 times more aware of the danger signs of childbirth than those who had labor at home, which is mainly due to the information given by the medical staff. Another study in Australia also supported that increasing the coverage of prenatal care and the education level of women elevates mothers' knowledge about the symptoms of childbirth risks. In another study, it was observed that the level of awareness was significantly associated with age, the level of education, employment status, and monthly household income of pregnant women (28). Therefore, the importance of raising awareness in pregnant women has been indicated previously, and the need for interventions to increase awareness is, today, felt more than ever.

Parsa et al. used a mobile application to educate pregnant women on pre-eclampsia. The results of this study emphasized the positive and significant effect of educational interventions on pregnant women's awareness about the symptoms of preeclampsia. This would reduce the risk of further serious complications of preeclampsia. Other studies also showed increased awareness of pregnant women undergoing educational intervention in the field of preeclampsia and gestational hypertensive disease (24–26, 29).

While education increases the ability of women to recognize pre-eclampsia and prevent related complications, being more informed is not associated with anxiety exacerbation (24). Pregnant women were very satisfied with receiving a pamphlet containing information on lethal potential of pre-eclampsia because their knowledge was increased without worsening the anxiety during the critical period of pregnancy (27). An HDP pamphlet developed patients' knowledge about future risks of health and pregnancy in a population of women with recent HDP diagnosis with the anxiety level remained unchanged. This knowledge that lasted at least for 1 month also elevated the perception of long-term heart disease. The authors concluded that lifestyle behavior is changed toward decreasing cardiovascular risk in this way (28).

It has been previously shown that maternal knowledge and awareness strongly affect the health of the child, which can be partly attributed to differences in prenatal care and reduction of adverse delivery outcomes (32). It is better to initiate educational programs as early as possible in pregnant women, and it should maintain for at least 3 months after partum (26). Different studies have investigated the effects of educational programs on pregnant women with different characteristics. Pregnant women of 20 to 32 weeks were recruited for receiving an educational tool in one study (24). Another study included postpartum women between 4 weeks and 18 months for educational intervention (28). A sample of 100 pregnant women who were hospitalized for suspected or proven pre-eclampsia was studied in the other study (27). In some studies, the duration of intervention was a 1-month period (11), whereas a 2-h educational session on pre-eclampsia was given to high-risk pregnant women in another study (26). With the implementation of educational programs, the health status of mothers and their babies are improved due to appropriate actions. This shows the importance of educational programs to reduce the risks and complications around pregnancy (26). However, some hurdles are identified that hamper effective use of health information during pregnancy, such as mistrust between patients and health care providers, and lack of suitable communication between them due to bad attitude of the latter (33).

One important strength of the present study is that it is the first systematic review on the effect of educational intervention on knowledge of pregnant women's knowledge about HDP. However, since the studies were conducted in three Asian countries and Canada, it is not possible to generalize the results to other populations and ethnicities.

## Conclusion

Altogether, the included studies showed that educational intervention strategies have a positive and significant impact on increasing the awareness of pregnant women about hypertensive disorders of pregnancy, which may help to reduce the severe complications caused by the disease. Future RCTs may compare the impact of various types of educational interventions on pregnant women.

## Data Availability Statement

The original contributions presented in the study are included in the article/supplementary material, further inquiries can be directed to the corresponding author/s.

## Author Contributions

KG and MD performed the search. AA and SK performed first screening. MS and HG performed second screening. KG and YS evaluated risk of bias. NN and MK drafted the manuscript. AK and SH revised the manuscript. ND designed, critically revised, and supervised the course of drafting the article. All authors contributed to the article and approved the submitted version.

## Conflict of Interest

The authors declare that the research was conducted in the absence of any commercial or financial relationships that could be construed as a potential conflict of interest.

## Publisher's Note

All claims expressed in this article are solely those of the authors and do not necessarily represent those of their affiliated organizations, or those of the publisher, the editors and the reviewers. Any product that may be evaluated in this article, or claim that may be made by its manufacturer, is not guaranteed or endorsed by the publisher.
